# Glaucoma Research in Transition: Mechanistic Insights, Novel Therapies, and Digital Frontiers

**DOI:** 10.3390/biomedicines13102491

**Published:** 2025-10-13

**Authors:** Wei-Ting Yen, Da-Wen Lu

**Affiliations:** 1 Department of Ophthalmology, College of Medicine, National Defense Medical University, Taipei 11490, Taiwan; jimmyyen59@gmail.com; 2 Department of Ophthalmology, Tri-Service General Hospital, Taipei 11490, Taiwan

Glaucoma continues to be one of the primary causes of irreversible blindness worldwide [1], and despite remarkable progress, its management continues to present significant challenges. The second edition of the Special Issue “Glaucoma: New Diagnostic and Therapeutic Approaches” brings together 13 contributions, spanning basic science, surgical innovations, artificial intelligence, and population-based perspectives. Together, these works highlight the evolving landscape of glaucoma research and underscore future directions needed to improve patient outcomes.

Emerging evidence highlights the role of neuroinflammation in glaucomatous optic neuropathy. Sato et al. demonstrated that microglial activation drives retinal ganglion cell damage in a rat ex vivo acute glaucoma model, suggesting that modulation of inflammasome pathways could represent a promising neuroprotective strategy [2]. Such mechanistic studies complement the long-recognized emphasis on intraocular pressure (IOP) control by pointing to the importance of targeting neurodegenerative pathways [3,4].

The therapeutic landscape continues to expand with novel surgical devices. Dierse et al. reported that Preserflo-MicroShunt implantation effectively lowered IOP and reduced medication burden, while maintaining endothelial cell density over one year [5]. These findings align with broader efforts to refine minimally invasive glaucoma surgery (MIGS), which has transformed the risk–benefit calculus in glaucoma surgery [6,7]. Long term, real-world studies will be essential to define optimal patient selection and durability of effect.

Artificial intelligence has become a central focus in modern ophthalmology. Ling et al. conducted a systematic review and meta-analysis, concluding that deep learning models achieve robust performance for both glaucoma detection and progression prediction [8]. The integration of multimodal clinical and imaging data holds the potential to move beyond structural or functional assessments alone, toward comprehensive predictive models [9,10,11]. Large-scale, multicenter validation will be crucial to ensure clinical translation and generalizability across populations [12,13].

The works presented in this Special Issue illustrate the breadth of contemporary glaucoma research, spanning from basic mechanisms to digital innovations. Despite this progress, critical gaps remain. Reliable biomarkers of progression, validated neuroprotective interventions, and robust multimodal AI models capable of predicting both disease progression and surgical outcomes are still urgently needed. Addressing these challenges will require large-scale, multicenter prospective studies to ensure clinical validity and generalizability.

By bringing together studies in neuroinflammation, surgical advances, and artificial intelligence, this Special Issue highlights the dynamic and multidisciplinary nature of glaucoma research. We extend our gratitude to all contributors, reviewers, and editorial staff, and we hope this collection will not only inform current practice but also inspire further innovation toward the shared goal of preventing blindness from glaucoma.
